# Impact evaluation of scripted lesson plans for HIV-related content in a life orientation curriculum: results from two provinces in South Africa

**DOI:** 10.1186/s12889-020-09640-2

**Published:** 2020-10-14

**Authors:** Ilene S. Speizer, Mahua Mandal, Khou Xiong, Ndinda Makina, Aiko Hattori, Darryn Durno

**Affiliations:** 1 Carolina Population Center, University of North Carolina at Chapel Hill, 123 W. Franklin St., Chapel Hill, NC 27516 USA; 2 Department of Maternal and Child Health, Gillings School of Global Public Health, University of North Carolina at Chapel Hill, Chapel Hill, NC USA; 3 SADC Research Centre, Cape Town, South Africa, USA

**Keywords:** HIV prevention, School-based programming, Pregnancy prevention, South Africa, Genital herpes

## Abstract

**Background:**

Young people under age 25 years are a key population at risk of unintended pregnancies, HIV and other sexually transmitted infections. School-based programming, focusing on youth under 17 years is strategic given that many in this age group are in school or are required to be in school and spend a considerable amount of their time at school. Prior evaluations of school-based HIV prevention programs for young people often employed weak study designs or lacked biomarkers (e.g., HIV or STI testing) to inform outcomes.

**Methods:**

This study used longitudinal data collected in 2016 from a cohort of grade-8 girls from Mpumalanga and KwaZulu-Natal Provinces in South Africa. We followed them for 2 years to examine the impact of the South African Department of Basic Education’s revised scripted lesson plans for the HIV and sexual content of a “life orientation” curriculum on knowledge, attitudes, condom use behaviors, pregnancy incidence, and genital herpes incidence. Schools were randomized to intervention and control arms. Multivariable analyses were undertaken using hazard modeling for incidence-based outcomes (genital herpes and pregnancy) and generalized linear latent and mixed modeling for outcomes measured at each time period (knowledge, attitudes, and condom use).

**Results:**

At end line, 105 schools were included from the two provinces (44 from Mpumalanga and 61 from KwaZulu-Natal). Fifty-five were intervention and fifty were control schools. A total of 2802 girls were surveyed at both time periods (1477 intervention and 1325 control). At baseline, participating girls were about 13.6 years; by end line, they were about 2 years older. Longitudinal data demonstrated few differences between intervention and control groups on knowledge, attitudes, condom use, genital herpes, and pregnancy experience. Monitoring data demonstrated that the program was not implemented as intended. Our results demonstrated 7% incidence of genital herpes in the two-year follow-up period indicating sexual risk-taking among our cohort.

**Conclusions:**

We did not find significant effects of the revised life orientation curriculum on key outcomes; however, this may reflect poor implementation. Future HIV prevention programs for young people need to be implemented with fidelity to ensure they meet the crucial needs of the next generation.

Trial Registration: This study has been registered at ClinicalTrials.gov. The trial registration number is: NCT04205721. The trial was retrospectively registered on December 18, 2019.

## Background

Young people under age 25 years are a key population at risk of unintended pregnancies and acquiring HIV and other sexually transmitted infections (STIs). They often lack access to effective contraceptives, including condoms, because of the social stigma of attaining a contraceptive method or a condom, lack of knowledge, and other factors [1, 2]. Globally, about one-third of new HIV infections in 2018 were among young people ages 15–24 years, and eastern and southern Africa accounted for the largest number of new infections [3]. In a meta-analysis of 18 sub-Saharan African countries, STIs other than herpes simplex virus type 2 (HSV-2) were found to be more common among young people ages 15–24 than among their older counterparts [4]. Additionally, descriptive analysis of Demographic and Health Survey data from 29 countries demonstrated that young women ages 15–19 years reported a greater percentage of pregnancies as unintended (37%) than did women ages 20–39 years (25–31% unintended) [5]. Addressing sexual and reproductive health (SRH) needs is a vital part of supporting young people’s overall health and well-being.

South Africa has one of the highest HIV incidence and prevalence rates among young people [6]. The 2016 South Africa Demographic and Health Survey found that HIV prevalence was 11.6% (95% CI: 8.7–14.6) among females ages 15–24 years and 3.4% (95% CI, 1.4–5.5) among males ages 15–24 [7]. In 2017, the annual incidence of HIV among young people ages 15–24 years was 1.5% (95% CI, 1.31–1.71) among females and 0.49% (95% CI, 0.27–0.71) among males [8]. The incidence for females ages 15–24 years was higher than females in other age groups, indicating the importance of targeting this group with HIV prevention. Further, recent STI testing among youth ages 15–24 years in KwaZulu-Natal Province, South Africa, demonstrated high prevalence of chlamydia (5% among males and 11% among females) and HSV-2 (17% among males and 29% among females) [9]. In a 2012 national survey, 83% of females ages 15–19 years were sexually active; 43.7% of those sexually active reported current contraceptive use [10]. The large percentage of young females not using contraception are at risk of unintended pregnancy. These findings indicate the importance of undertaking comprehensive SRH programming for young people in South Africa, with a focus on locations with higher incidence and prevalence of these SRH outcomes.

Sexual and reproductive health education and behavior change programs have been implemented through mass media, community-based programming, youth-friendly service delivery, and schools, among other strategies [11, 12]. School-based programming focused on youth ages 17 years and below can be an effective vehicle to provide SRH education and promote positive behaviors given that many young people in this age group are in school or are required to be in school and spend a considerable amount of their time at school. Recent meta-analyses of school-based HIV and SRH prevention programs have demonstrated mixed results of their effect on prevention of adolescent pregnancy, sexually transmitted infections, and HIV in the United States [13, 14], in low- and middle-income countries (LMIC) [15], and in global reviews [16]. Based on these reviews, it is not possible to recommend specific approaches that work for future pregnancy, STI, and HIV prevention programming for young people. The approaches used for the evaluations of pregnancy, STI, and HIV prevention programming use different outcomes and study methods. For example, one of the systematic reviews focused on LMIC included self-reported SRH outcomes (e.g., knowledge, attitudes, sexual initiation, condom use, and number of partners) [14], and another global review that identified eight eligible studies focused on health status outcomes such as incidence and prevalence of HIV, STIs, or pregnancy [16]. Furthermore, while most of the meta-analyses included randomized controlled trials (RCTs) and other study designs that were able to attribute impact to a program, many studies within the meta-analyses were ranked as having low to moderate levels of evidence [13–16]. Finally, the studies from LMIC that examined self-reported outcomes generally showed that the school-based programs increased knowledge, self-efficacy, and condom use; however, these evaluations had weaker study designs [15] than the small number of studies that used RCTs and measured health status outcomes [16].

Results of these meta-analyses demonstrated little significant contribution of school-based programming on HIV incidence or prevalence, HSV-2 prevalence, and pregnancy [13, 14, 16]. Studies using biomarkers (e.g., dried blood spots or a blood draw to measure STI, HIV or pregnancy) are often more rigorous because they avoid the biases that come from self-reported study outcomes. Some of the null effects from studies with biomarker outcomes may be the result of small sample sizes.

In a recent study from South Africa undertaken in collaboration with the Department of Education, Visser [17] sought to examine behaviors of young people to help inform school-based programs for the future. The author surveyed school-going young people in grades 5–12 in four provinces adversely affected by HIV and AIDS. The author demonstrated that in the sample of young people ages 14–18 years, 49.4% of boys and 30.5% of girls reported that they had ever had sex. About a third of the sexually active boys in this age group reported multiple partners in the past 3 months. Similarly, previously published baseline data for the evaluation that is the focus of the present paper [18] demonstrated that among young people in grade 8 in KwaZulu-Natal and Mpumalanga Provinces (average age 13.5 years), about 20% of boys and 9.5% of girls self-reported being sexually experienced. Further, in the KwaZulu-Natal and Mpumalanga sample of young people who had ever had sex, about three-quarters of males and 70% of females reported using condoms at last sex, and 37% of sexually experienced girls from KwaZulu-Natal and 28% from Mpumalanga reported that they had ever been pregnant [18]. These South African findings demonstrate that despite long-term efforts to address HIV education in schools in South Africa [19], young people are still engaging in high-risk sexual behaviors. Therefore, there is a need to connect improving knowledge, which is relatively easy to do in the school setting, with changing behaviors in the long term.

This study fills gaps in our earlier knowledge of HIV and SRH programming in South Africa by using a randomized cluster design following a longitudinal cohort of young people in grade 8 in 2016 for 2 years and measuring outcomes using biomarkers (genital herpes and HIV) and self-reported behaviors (knowledge, attitudes, and condom use). The study was commissioned to evaluate the novel HIV Life Orientation (LO) program developed by the South Africa Department of Basic Education that used scripted lesson plans and supporting activities for the provision of SRH content.

### Life orientation intervention

This evaluation focused on the HIV and SRH content of the Government of South Africa LO Curriculum. In 2010, the Department of Basic Education (DBE) undertook assessments of their LO program and learned that while young people who participated in the program had improved knowledge and attitudes, the program was not being implemented uniformly [19]. To address this concern, DBE, with support from the United States Agency for International Development (USAID) and the United States President’s Emergency Plan for AIDS Relief (PEPFAR), developed scripted lesson plans (SLP) to strengthen the SRH content and standardize implementation across schools; these SLP were paired with supporting activities to address fidelity in the curriculum’s delivery.

The content of the SLPs included active lessons that focused on the following six core messages: (1) encouraging young people to say no to sex; (2) supporting young people to recognize that they have the right to say no to sex in any situation; (3) clarifying that if they choose to have sex, to use a condom every time; (4) promoting the importance of being faithful to one partner at a time; (5) specifying the need to get tested for HIV and other STI if having sex; and (6) recognizing that both men and women are responsible for preventing pregnancy, HIV, and other STIs. The SLP were integrated in the LO program, with most of the lessons offered in the first half of the school year. There are eight lessons for grade 8, 11 for grade 9, and 10 for grade 10. Participants in the control schools still covered the above topics, but with the standard curriculum and not with the upgraded SLP.

A key component of the intervention was that all LO teachers were trained on the SLP prior to implementation. Since 2015, USAID/PEPFAR provided technical support to DBE for program rollout in priority provinces and districts that have the highest HIV incidence and prevalence. Technical support included educator training on the new SLP and considerations of approaches for scale-up beyond the initial districts. Support for the first phase of implementation and testing was led by Education Development Center (EDC), with funding from USAID. In the intervention arm, life orientation teachers attended a 4–5 day DBE- and EDC-led training to use the SLP to teach the sexuality and HIV prevention content of the LO program. In the control arm, teachers followed the existing LO curriculum with no additional training. Each year, a new teacher training program was implemented, because of the high turnover of teachers who facilitated LO classes in the schools.

Through discussions with EDC and as indicated in monitoring data received from EDC at the end of the program implementation period, it became clear that delays in getting DBE approvals for release of the grade-10 curriculum resulted in incomplete implementation of the program in 2018. This has the potential to affect the impact results and is addressed in the discussion section.

## Methods

### Study design

This study used a stratified cluster randomized sampling approach. The evaluation of the program covered five education districts in two provinces (Mpumalanga and KwaZulu-Natal) with a high prevalence of STIs and pregnancy, as identified by the USAID mission in South Africa (USAID/SA) and the DBE. Target schools with students in grades 8–10 were located in the three lowest socioeconomic status (SES) quintiles. The sampling frame was constructed at baseline from a list of schools provided by the DBE and confirmed with the provincial-level departments of education; this list had information on the schools’ locations and measures of the SES of the catchment students. We then stratified the confirmed frame by the five education districts in the two provinces: Bohlabela and Gert Sibande Districts, in Mpumalanga, and King Cetshwayo, Pinetown, and Umlazi Districts, in KwaZulu-Natal. We selected a stratified random sample of schools whereby the number of selected schools within each district was proportional to the number of eligible schools in the district within each province, so as to reflect the composition of the target population in each province. Then, within each district, we randomly assigned the selected schools either to the intervention or control arm. In total, we randomly selected 115 schools and assigned 58 as intervention schools (23 in Mpumalanga and 35 in KwaZulu-Natal) and 57 as control schools (22 in Mpumalanga and 35 in KwaZulu-Natal).^1^

### Target population

The target population for the impact evaluation was female grade 8 students in 2016 who would be followed for a two-year period (in and out-of-school) to examine changes in their biological outcomes (HSV-2 and pregnancy experience) and their self-reported sexual and reproductive health outcomes. The focus for the impact evaluation was on female students due to their higher prevalence of HSV-2 (and HIV) and thus smaller required sample size compared to male students. Additionally, only females could directly experience pregnancy. To complement the longitudinal impact evaluation findings, data were also collected from cross-sectional samples of male students in grade 8 in 2016, grade 9 in 2017 and grade 10 in 2018; this male sample is not discussed further but details can be found elsewhere [20, 21].

### Sampling and response rates

At baseline, the objective was to survey all grade 8 students in the study schools. The goal of the sample size calculation was to power the statistical analysis of the primary outcome—that is, the composite measure of incidence of HSV-2 or pregnancy in a two-year period (see below) among a cohort of grade-8 female students. We designed the sampling plan to recruit 2500 female students in grade 8 in each of the two arms (5000 female students in total) from 115 schools. We based sample size calculations on assumptions and specifications of sampling parameters. First, we specified the minimum detectable change in the primary outcomes based on the assumed incidence rate of HSV-2 or pregnancy of 0.04 in the intervention arm versus 0.08 in the control arm over two school years at the significance level (α) of 0.05 (two-sided). Next, we adjusted the sample size for the following: (1) design effect, to account for elevated standard errors in a cluster sample design; (2) baseline prevalence of the primary outcome, to account for loss of units available to estimate the incidence rate^2^; and (3) nonresponse of schools and female learners. We approximated the design effect from clustering as 1 + ICC×(*M*− 1), where ICC is intra-cluster correlation and *M* is the average cluster size [22]. We assumed an ICC of 0.03 and an average of 50 female students per school, implying a design effect of 2.47. Next, we assumed prevalence at 1% for HSV-2 and 0% for pregnancy at baseline. Finally, we accounted for potential nonresponse in schools (15 out of 115 schools) and assumed a response rate of 70% for grade-8 female learners at baseline. With a total sample size in both arms of 3500 female students successfully interviewed at baseline (or 5000 female students recruited for interviews with an assumed response rate of 70%), we estimated a statistical power (1-β) of 88%. The sampling plan assumed that larger schools would include more participants and smaller schools would include fewer participants. A total of 3583 grade 8 female students were surveyed at baseline [20].

At follow-up in 2018, we sought to interview all girls who had been in grade 8 at baseline. To ensure a valid estimate of intervention effects, baseline female students were followed even if they dropped out of school after the 2016 baseline survey. This involved determining whether cohort participants were still enrolled in the school they had attended in 2016, were no longer in school, or had moved since 2016. A midline survey was undertaken in 2017 which helped to identify girls who had moved within 1 year of participating in the baseline survey. Further tracing mechanisms were employed in 2018, including following up on alternative contacts provided in the contact list at the time of the 2016 survey. The contact list included addresses and contact numbers of relatives, social media profiles, and group memberships such as church or youth groups. School-level tracing activities included enquiring about a former learner’s whereabouts with teachers and pupils. A dedicated tracing unit first telephoned all contact numbers and, if these proved unsuccessful, undertook physical visits to last known addresses and areas where girls had resided. At follow-up in 2018 we found a total of 2802 of the girls that were in grade 8 in 2016 (78.2% response rate from baseline respondents). In Mpumalanga, we found 87.4 and 85.8% of the intervention and control groups, respectively; in KwaZulu-Natal, these percentages were lower at 72.4 and 71.0%, respectively. Of note, there were about 300 grade-8 students for which we did not have names or contact information from baseline; these girls may have still been in their baseline schools in 2017 and 2018, but it was not possible to link them. After excluding these students, the response rates were greater than 90% in Mpumalanga and 78% in KwaZulu-Natal. See Table 1 for details of the 2016 and 2018 samples, including the response rates.

**Table 1:** Characteristics of sample of girls included in longitudinal cohort at baseline (2016) and 2 years later (end line), Mpumalanga and Kwa-Zulu Natal, South Africa

	Baseline Full Sample		Baseline (Grade 8) Matched Cohort		End Line Matched Cohort	
	Intervention	Control	Intervention	Control	Intervention	Control
	Mpumalanga					
Response rate at end line (%)						
Full sample	na	na	na	na	87.4%	85.8%
Full sample removing those without contact information	na	na	na	na	93.1%	91.3%
**Demographics**						
Mean age	13.61	13.55	13.55	13.46	15.58	15.40
Orphanhood (%)						
Non-orphan	60.01	63.15	61.27	64.03	59.96	62.03
Single orphan	29.83	27.46	29.30	26.94	31.64	29.79
Double orphan	10.17	9.39	9.43	9.03	8.40	8.18
Religion (%)						
Christian	80.96	79.22	82.14	79.75	90.50	92.93
Traditional	12.40	14.05	11.50	14.24	7.18	5.01
Other	6.64	6.73	6.36	6.00	2.32	2.06
Food security (%)						
No days without food in past 3 days	75.08	76.44	75.58	77.81	80.00	83.70
Any days without food in past 3 days	24.92	23.56	24.42	22.19	20.00	16.30
HIV-positive person in household (%)						
Someone is HIV positive	12.61	9.70	11.23	8.89	16.08	12.55
No one is HIV positive	58.63	59.94	59.55	60.22	56.12	58.43
Don’t know if anyone in household is HIV positive, %	28.76	30.36	28.75	30.60	27.79	29.02
**Number of observations**	**833**	**785**	**726**	**668**	**726**	**668**
	KwaZulu-Natal					
Response rate at end line (%)						
Full sample	na	na	na	na	72.4%	71.0%
Full sample removing those without contact information	na	na	na	na	78.6%	79.0%
**Demographics**						
Mean age	13.55	13.62	13.47	13.53	15.48	15.52
Orphanhood (%)						
Non-orphan	54.97	57.55	55.99	58.34	55.76	56.10
Single orphan	31.64	30.40	31.47	29.38	33.29	31.10
Double orphan	13.39	12.05	12.55	12.29	10.94	12.80
Religion (%)						
Christian	59.05	55.14	60.85	53.71	61.80	58.04
Traditional	26.89	32.16	26.08	34.62	29.47	33.76
Other	14.07	12.70	13.07	11.67	8.73	8.20
Food security (%)						
No days without food in past 3 days	69.13	66.70	70.36	68.83	76.79	74.66
Any days without food in past 3 days	30.87	33.30	29.64	31.17	23.21	25.34
HIV-positive person in household (%)						
Someone is HIV positive	16.11	13.99	15.38	14.03	21.92	21.39
No one is HIV positive	57.02	60.34	58.91	60.90	47.58	49.03
Don’t know if anyone in household is HIV positive, %	26.87	25.67	24.86	24.67	30.51	29.57
**Number of observations**	**1040**	**925**	**751**	**657**	**751**	**657**

### Survey tool

The baseline survey was conducted between August and November 2016; the midline survey was conducted between August and September 2017; and the end line survey was conducted between August and November 2018. The survey instrument administered at baseline, midline, and end line was developed in consultation with stakeholders from the DBE, EDC and their consortium partners, and USAID. It consisted of the following topics: demographics and household composition; connectivity to caregivers; school attendance and performance; aspirations and expectations about the future; risk perceptions; sexual behaviors; and participation in and perceptions of the LO curriculum. Questions included in the survey were taken or adapted from other surveys in South Africa or from validated scales (e.g., the Gender Equitable Men scale [26]); or were developed based on the content of the LO curriculum (e.g., knowledge and attitude questions). Details on the sources of key survey items can be found in Tables 2 and 3.

**Table 2:** Measures used to create scales for secondary outcomes

Variable	Measurement Approach	Cronbach’s Alpha	Score Creation
Knowledge score			
• You can usually tell if someone has HIV and AIDS by the way they look.^a^	True, false, don’t know (coded 1 if correct; zero otherwise)	0.7019	Calculated the mean correct responses (range 0–1) – higher score equals greater knowledge
• If you have a sexually transmitted infection (STI) you will definitely know because you will see/feel symptoms.^a^			
• Not all sexually transmitted infections are curable.^a^			
• Oral sex has no risk for STIs.^a^			
• When used correctly and consistently, condoms protect you from all STIs.^a^			
• If a mosquito bites you it can infect you with HIV.^b^			
• You can get HIV from kissing a person who is HIV positive.^b^			
• A woman who is pregnant can do nothing to prevent her baby from being born with HIV.^b^			
Attitude score^c^			
• Being exposed to the saliva of a person with HIV or AIDS	Have fear of this (coded 1), Do not have fear of this or Do not know (coded zero)	0.6381	Calculated mean attitude score (range 0–1) – higher value reflects worse attitudes
• Being exposed to the sweat of a person with HIV or AIDS			
• Sharing eating utensils with a person who has HIV or AIDS			
• Physically caring for a person living with HIV or AIDS			
• HIV is a punishment from God.			
• HIV and AIDS are punishment for bad behavior.	Agree (coded 1) Disagree (coded zero)		
• It is women prostitutes who spread HIV in the community.			
• It is men prostitutes who spread HIV in the community.			
• People with HIV are promiscuous.			
• I would be ashamed if I were infected with HIV.			
• I would be ashamed if someone in my family had HIV and AIDS.			
Self-efficacy score^d^			
**How confident are you that…**			
• If your friends were having sex with their boyfriends or girlfriends, could you refuse to start having sex if you did not want to?	Yes (coded 4)A little bit (coded 3)I’m not sure (coded 2)No (coded 1)	0.7237	Calculated mean self-efficacy score (range 1–3) – higher score means more self-confident
• If your boyfriend was pressuring you to have sex and you did not want to, could you refuse to have sex with him?			
• If your boyfriend or girlfriend was asking you to have sex, would you be able to talk about condoms with him or her?			
• Do you feel confident you could refuse to have sex even if someone offered you a meal, gifts, money, or favors in exchange?			
Gender norms score (from GEM scale^e^)			
• It is the man who decides when to have sex.	Agree a lot,Somewhat agree,Do not agree at all (Items standardized to have positive values - coded 1 for less equitable response and 3 for more equitable response)	0.7579	Calculated mean gender equitable score (range 1–3) higher score means more gender equitable norms
• Men are always ready to have sex.			
• Women are always ready to have sex.			
• Men need sex more than women do.			
• A man needs other partners even if things with his wife/partner are fine.			
• A woman needs other partners even if things with her husband/partner are fine.			
• You don’t talk about sex, you just do it.			
• A woman should not initiate sex.			
• A woman who has sex before she is married does not deserve respect.			
• A man who has sex before he is married does not deserve respect.			
• Women who carry condoms on them are loose.			
• Men who carry condoms on them are loose.			
• In my opinion, women can suggest using condoms just like a man can.			
• A couple should decide together if they want to have children.			
• It is only the woman’s responsibility to avoid getting pregnant.			
• If a man gets a woman pregnant the child is the responsibility of both.			
• It is important that a father is present in the lives of his children, even if he is no longer with the mother.			

Note: Cronbach’s alpha presented for endline cohort sample

^a^Knowledge items created based on LO curriculum; ^b^Knowledge items that come from the HPTN-068 study questionnaire [23]; ^c^Attitude items came mostly from the Nyblade and MacQuarrie Stigma and Discrimination Scale [24]; ^d^The self-efficacy items come from Y-HAPP T2 [25] and items developed based on the LO curriculum content; ^e^From the Gender Equitable Men Scale [26]

**Table 3:** Descriptive results of key intermediate outcomes by place of residence and intervention group

	Mpumalanga				KwaZulu-Natal			
	Baseline (Grade 8) Matched Cohort		End Line Matched Cohort		Baseline (Grade 8) Matched Cohort		End Line Matched Cohort	
	Intervention	Control	Intervention	Control	Intervention	Control	Intervention	Control
Knowledge score (mean)	0.36	0.35	0.43	0.41	0.29	0.28	0.39	0.38
Attitude score (mean)	0.45	0.42	0.40	0.40	0.41	0.44	0.40	0.41
Self-efficacy score (mean)	2.72	2.80	3.18	3.21	2.82	2.78	3.13	3.10
Gender score (mean)	2.21	2.21	2.37	2.37	2.35	2.37	2.45	2.44
Learning in Life Orientation class (mostly true and very true) (mean)^a^								
The things we learn about gender roles, sexuality, and HIV in the LO class are similar to what I experience in my life.	2.92	2.90	2.83	2.75	2.62	2.60	2.63	2.55
I have learned a lot about sexuality and HIV-related topics in my LO class.	3.41	3.29	3.45	3.38	3.15	3.06	3.37	3.28
I am able to apply some of the things I have learned about gender roles, sexuality, and HIV in the LO class to my personal life.	3.02	3.06	3.01	2.97	2.85	2.81	2.85	2.77
Score of three (Cronbach’s alpha = 0.6452)	3.12	3.08	3.09	3.03	2.87	2.82	2.94	2.87
Participate in Life Orientation class (mean)^a^								
Level of participation in class discussions in LO lessons?	2.54	2.51	2.55	2.48	2.47	2.46	2.53	2.51
Frequency ask the teacher questions during your LO lessons?	2.08	2.09	2.06	1.99	2.09	2.06	2.02	1.98
Extent listen to what the teacher is teaching you in LO lessons?	2.79	2.74	2.73	2.71	2.72	2.70	2.73	2.69
How motivated are you to learn about LO?	2.73	2.72	2.71	2.73	2.71	2.66	2.75	2.68
Time and effort to do assignments or study for LO tests?	2.57	2.55	2.64	2.64	2.55	2.47	2.59	2.53
Score of five (Cronbach’s alpha = 0.6967)	2.54	2.52	2.54	2.51	2.51	2.47	2.52	2.48
Perspectives of Life Orientation teacher (mean)^a^								
LO teacher encourages students to learn the material	3.07	2.86	3.20	3.16	3.14	3.11	3.18	3.08
LO teacher understands the material that he/she presents	3.33	3.30	3.31	3.30	3.36	3.28	3.34	3.23
LO teacher wants all students to feel respected	3.69	3.63	3.65	3.60	3.49	3.45	3.54	3.52
Score of three (Cronbach’s alpha = 0.7243)	3.36	3.27	3.38	3.35	3.33	3.28	3.35	3.28
HIV test in the past 12 months (%)	26.10	30.70	44.40	36.90	31.50	35.00	36.10	32.40
Clinic visit for SRH in past 12 months (%)	27.79	28.81	49.78	43.46	33.0	28.75	38.35	39.10

^a^All questions asked on a four-point scale (not true to very true) and scores are based on the mean across the items included. SRH: sexual and reproductive health. Life Orientation questions were adapted from a recent school-based survey by HEARD in Kwa-Zulu Natal, South Africa [25]. Note: Cronbach’s alpha presented for endline cohort sample

Professional translators translated the surveys into local languages: English, Sepedi, SiSwati, xiTsonga, and IsiZulu. The surveys were uploaded on tablets using Open Data Kit (ODK); participants could read or listen to each question to complete the self-administered survey.

Participants in the intervention and control arms were also asked to provide dried blood spots (DBS) to measure the biological outcomes (see details of consent below). Dried blood spots were collected by study nurses at baseline in 2016 and again at end line in 2018. Following DBS data collection, study nurses undertook a screening questionnaire of participants and referred to the local public health center any participant potentially reporting symptoms of STI. All participants received an envelope with a thank-you letter; however, those who were being referred had an additional referral notice in their sealed envelopes. Biomarkers collected in 2016 were stored at − 80 degrees Celsius at the University of Pretoria for analysis after end line data collection. All biomarker samples collected in 2018 were tested for HSV-2. If a participant’s sample tested positive for HSV-2 in 2018, her corresponding 2016 sample was tested for HSV-2 to determine whether her HSV-2 infection occurred between 2016 and 2018 (i.e., incident HSV-2 infection). Additionally, all of the biomarkers collected in 2018 were tested for HIV, which permitted assessment of HIV prevalence among the cohort of female students interviewed in 2018.^3^ All samples were sent to Global Clinical and Viral Laboratory (SA) in KwaZulu-Natal for specimen testing using IgG testing for HSV-2 and Elisa screening and Elisa confirmation testing for HIV. Pregnancy self-reports were obtained at each survey wave.

### Outcomes

At the end of 2018, the team measured the impact of the new program by comparing the incidence of HSV-2 or pregnancy (a composite variable), and HIV prevalence among the cohort of grade-8 female students enrolled in the selected schools in 2016 in the intervention and control arms. The key biological outcomes, incidence of HSV-2 and prevalence of HIV, came from the dried blood spots. We examined self-reported pregnancy experience and the timing of first pregnancy experience at baseline and at end line to determine if there was an incident pregnancy in the follow-up period. The composite outcome of incidence of HSV-2 or pregnancy was created from the above single outcomes and coded one if a participant experienced either a new HSV-2 infection or a new pregnancy between baseline and end line; the composite incidence measure was used because the incidence was expected to be low for each outcome.

We included in our analysis other self-reported behavioral outcomes measured at baseline and end line including HIV testing in the past 12 months (yes vs. no) and visiting a clinic for SRH services in the past 12 months (yes vs. no). We also included self-reported sexual experience in the analysis. This was used to learn the incidence of sexual activity in the follow-up period based on the reports from baseline compared to end line. Cases of inconsistencies (i.e., a girl report having ever had sex at baseline and not at end line) were set to missing and excluded from the analysis. The last self-reported behavioral outcome was the number of sexual partners in the past 12 months; this was examined in the multivariate analyses among only those who reported that they had ever had sex at end line.

Intermediate outcomes including knowledge, attitudes, and self-efficacy were also assessed at baseline and end line for this analysis. Details on the items measured in the survey, the response options, the Cronbach’s alpha, and the score creation are included in Table 2. For each scale, we summed the responses to the items and divided by number of items to create a knowledge score (higher score equals higher knowledge), an attitude score (higher score means worse attitudes, i.e., more fear), a self-efficacy score based on saying yes to confidence on the four items, and a gender norms scale based on the Gender Equitable Men Scale (items are standardized so that they have the same meaning, positive values are summed, and a higher score represents higher support for gender equitable norms) [26]. We also created scores based on responses about the participants’ perspectives on the life orientation curriculum they used in school (SLP – intervention group; or standard of care – control group). Descriptive results for these life orientation variables appear in Table 3. Copies of the 2016 and 2018 survey tools are available at: https://dataverse.unc.edu/dataverse/cpc.

### Data analysis

We analyzed data using Stata statistical software version 15.1 (Stata Corp LP, College Station, Texas) by applying sampling weights and estimating cluster robust standard errors to account for the sampling design and nonresponse. We computed descriptive statistics and frequency distribution for all variables analyzed for the cohort sample. Analysis of the longitudinal sample employed multiple methods according to the outcome of interest. We analyzed outcomes that represent incidence between baseline and end line through the Cox proportional hazards model within the framework of survival analysis. The outcomes measuring incidence since baseline were ever tested for HIV; ever had sex; ever became pregnant; acquisition of HSV-2; and the composite indicator of ever became pregnant and acquired HSV-2. We measured these outcomes as ever occurred between baseline and end line. Data from individuals whose baseline status was negative for each outcome were analyzed. The focus of the analysis is on the estimated difference between the intervention and control schools in the cohort sample.

Knowledge, attitudes, and behaviors that were measured at each survey point and could vary across time were analyzed through generalized linear latent and mixed models (GLLAMM). The focus of the analysis is the difference in the change in the outcome across time between the intervention and control schools in the cohort sample (i.e., an interaction effect).

Finally, the prevalence of HIV at end line was analyzed through a logistic regression. The outcome of interest was the difference in HIV prevalence at end line between the intervention and control schools in the cohort sample.

The analysis was implemented as an intention-to-treat analysis, which addresses bias owing to self-selection in an intervention or control school. The following were control variables: age, orphanhood (not an orphan, single orphan, double orphan), having an HIV-positive person in the household, food insecurity (whether there were days without food in home in the past 3 days), religion (Christian, traditional, other), and district. The control variables are presented in Table 1. For the analysis, we use baseline control variables to minimize potential bias due to endogeneity.

### Consent procedures and ethics approval

At each round of data collection, all participating girls under the age of 18 years received written parental consent to be surveyed and each young person provided written assent. Participants were told that they could stop the survey at any time without any negative implications. Separate written parental consent and participant assent was employed for collection of biomarkers. The study protocol including the consent and assent procedures were approved by the University of Pretoria Faculty of Health Sciences Research Ethics Committee (Ref. No. 153/2016) and the University of North Carolina at Chapel Hill Institutional Review Board (IRB Number 15–3217). Enterprises University of Pretoria (Pty) Ltd. collected baseline data; SADC Research Centre collected midline and end line data.

## Results

Table 1 presents the characteristics of the full cohort of girls enrolled at baseline (2016) when they were in grade 8 (full sample), the matched grade-8 baseline sample (baseline matched cohort), and the characteristics of the matched cohort interviewed in 2018 (end line matched cohort) by province and study arm (intervention or control). Overall, the characteristics of the full sample and the matched sample in 2016 were similar; this suggests that the loss to follow-up was not related to these observed demographic characteristics. In Mpumalanga and KwaZulu-Natal at baseline, the mean age of the grade-8 girls in both provinces was about 13.5 years. By end line, as expected, the cohort had aged 2 years and the average age was about 15.5 years. No differences were found by intervention arm. In Mpumalanga, about four-fifths of the sample was Christian, and the remaining girls were traditional or another religion; by end line, the percentage reporting to be Christian increased to more than 90%. In KwaZulu-Natal, a greater percentage of surveyed girls reported a traditional religion (about 30%); little difference was observed by end line.

At baseline in Mpumalanga, a little less than two-fifths of girls were either single orphans (26–29%) or double orphans (9–10%). In KwaZulu-Natal, a slightly higher percentage of young people in grade 8 were single (29–31%) or double orphans (12–13%). By end line, the level of orphanhood increased slightly in both provinces; however, slightly fewer young people reported being double orphans at end line in Mpumalanga and in the intervention group in KwaZulu-Natal. At baseline, about one-quarter of girls in Mpumalanga and one-third in KwaZulu-Natal reported going any of the past 3 days without any food/food insecurity. This declined in both provinces in the intervention and control groups by end line. At baseline, about 10% of girls in Mpumalanga and about 15% of girls in KwaZulu-Natal reported that someone in their household was HIV positive (Table 1). By end line, as expected in these provinces with high HIV prevalence, this increased in both intervention and control groups in both provinces.

Table 2 presents the indicators used to create the intermediate outcomes and Table 3 presents the descriptive results of these outcome variables by province, time period, and intervention and control group. Table 3 demonstrates few differences in the knowledge, attitudes, self-efficacy and gender scores between grade 8 girls in intervention and control schools at baseline in both provinces. By end line, generally, scores for knowledge, self-efficacy, and the gender score had increased in both the intervention and control groups in both provinces, but no obvious pattern emerges for girls who were in the intervention arm. The attitudes score generally declines between baseline and end line, indicating more positive attitudes; however, declines were extremely small for this indicator. Also shown in Table 3 are the items used to create indicators related to the LO and their respective scores. In Mpumalanga, scores on what was learned in the LO class and participation in LO class stayed relatively stable or declined between baseline and end line. That said, the score for the perspective of the LO teacher between baseline and end line in Mpumalanga increased; this increase was observed in both intervention and control schools. In KwaZulu-Natal, where slight increases were observed between baseline and end line in the LO indicators, these increases were observed in both the intervention and control arms.

Slight improvements in the HIV testing and clinic visits are seen over time, particularly in Mpumalanga (Table 3). Increases appeared to be greater in the Mpumalanga intervention group than the Mpumalanga control group at end line, whereas there was a larger increase in the control group than the intervention group for SRH clinic visits in KwaZulu-Natal.

The percentage of girls who ever had sex increased in both arms and provinces between baseline and end line (Table 4). For example, in Mpumalanga, the percentage increased from about 8–9% to 32–33%, whereas in KwaZulu-Natal, the percentage of those who had ever had sex only increased to about 20%. In addition, the percentage of those who were ever pregnant in the full sample also increased over the follow-up period, from about 2% at baseline to 5–6% at end line. At baseline, about 2% of girls in Mpumalanga had genital herpes (HSV-2) whereas this value was 3.6% in KwaZulu-Natal. By end line, the percentage who had HSV-2 increased in both provinces, to about 10%. The prevalence of HIV at end line was about 5% in both provinces. Finally, Table 4 demonstrates that on average among sexually experienced girls, the number of sexual partners in the past year increased over time; the average number of sexual partners at end line was lower in the intervention groups in both provinces than in the control groups.

**Table 4:** Descriptive results of biological and behavioral outcomes by place of residence and intervention group

	Mpumalanga				KwaZulu-Natal			
	Baseline (Grade 8) Matched Cohort		End Line Matched Cohort		Baseline (Grade 8) Matched Cohort		End Line Matched Cohort	
	Intervention	Control	Intervention	Control	Intervention	Control	Intervention	Control
Ever sex (%)	9.30	8.20	31.70	33.00	8.90	6.20	21.30	19.00
Ever pregnant (%)	2.40	1.90	7.30	5.80	2.30	1.20	7.50	4.70
End line HIV (%)	na	na	4.80	5.80	na	na	5.20	4.30
HSV-2 (%)	2.60	2.20	11.70	9.00	3.60	3.60	9.30	11.10
HSV-2 or ever pregnant (%)	4.8	4.1	12.00	9.60	5.1	4.8	8.20	8.40
**Among those who ever had sex by end line:**								
Number of partners in the past 12 months^a^ (mean)	0.70	0.50	1.30	1.50	1.00	0.70	1.10	1.30

^a^Replaced two observations to missing for girls that had their number of partners in the last 12 months greater than 50 at baseline

Multivariate results of the analysis of the intermediate, behavioral, and health status outcomes with a focus on the intervention effect are presented for the full sample and stratified by study province in Table 5. Results for the full sample demonstrate that the intervention did not have a significant effect on the knowledge, attitudes, and LO outcomes. Significant effects on clinic visits were found such that in the full sample and in the stratified analyses, girls in the intervention group were significantly more likely to have had a recent HIV test at end line. Furthermore, in Mpumalanga, intervention girls were also significantly more likely than control girls to have had a recent SRH clinic visit.

**Table 5:** Multivariate adjusted longitudinal results of impact of intervention on intermediate and biological and behavioral outcomes for full sample and by place of residence

	Full Sample	Mpumalanga	KwaZulu-Natal
**Intermediate Outcomes**			
Knowledge score^a^	0.00 (0.012) (*p* = 0.743)	0.01 (0.012) (*p* = 0.308)	0.00 (0.015) (*p* = 0.967)
Attitudes score (high bad)^a^	0.00 (0.015) (*p* = 0.885)	0.00 (0.016) (*p* = 0.832)	0.00 (0.020) (*p* = 0.945)
Self-efficacy score^a^	−0.01 (0.042) (*p* = 0.805)	−0.01 (0.045) (*p* = 0.843)	−0.00 (0.052) (*p* = 0.980)
Gender score^a^	0.01 (0.017) (*p* = 0.680)	−0.00 (0.025) (*p* = 0.915)	0.01 (0.021) (*p* = 0.542)
Learn a lot in LO class (average)^a^	0.06 (0.043) (*p* = 0.178)	0.06 (0.044) (*p* = 0.211)	0.06 (0.054) (*p* = 0.293)
Participate in LO class (average)^a^	0.03 (0.023) (*p* = 0.252)	0.02 (0.025) (*p* = 0.461)	0.03 (0.029) (*p* = 0.318)
Perspective of LO teacher (average)^a^	0.04 (0.048) (*p* = 0.379)	0.00 (0.066) (*p* = 0.953)	0.06 (0.059) (*p* = 0.346)
Recent HIV test^a^	0.34 (0.146) (*p* = 0.018)	0.44 (0.228) (*p* = 0.054)	0.33 (0.179) (*p* = 0.065)
Recent SRH clinic visit^a^	0.07 (0.118) (*p* = 0.563)	0.26 (0.155) (*p* = 0.097)	0.02 (0.144) (*p* = 0.896)
**Biological and Behavioral Outcomes**			
Ever had sex^b^	1.02 (0.113) (*p* = 0.889)	1.06 (0.129) (*p* = 0.635)	0.99 (0.155) (*p* = 0.971)
Ever-pregnant^b^	1.55 (0.328) (*p* = 0.038)	1.42 (0.457) (*p* = 0.274)	1.64 (0.414) (*p* = 0.050)
End line HIV ^c^	1.14 (0.211) (*p* = 0.493)	0.76 (0.184) (p = 0.252)	1.33 (0.319) (*p* = 0.240)
HSV-2^b^	0.84 (0.184) (*p* = 0.428)	1.28 (0.267) (p = 0.240)	0.73 (0.204) (*p* = 0.260)
HSV-2 or ever-pregnant^b^	1.04 (0.178) (*p* = 0.797)	1.32 (0.235) (*p* = 0.123)	0.96 (0.213) (*p* = 0.850)
Number of partners in the past 12 months^a, d^	−0.21 (0.235) (*p* = 0.365)	−0.21 (0.351) (*p* = 0.546)	−0.22 (0.277) (*p* = 0.417)

Reported figures are estimated coefficients (SE) and significance level

All models control for age group, orphanhood, if there is an HIV-positive person in the household, food insecurity, religion, and districts

Sample sizes:

• All outcomes except HIV, HSV-2, number of partners – Total = 2802; KwaZulu-Natal = 1408; Mpumalanga = 1394 (some n's smaller due to missing values)

• HIV and HSV-2 analyses – Total = 2,6,84; KwaZulu-Natal = 1351; Mpumalanga = 1333

• Number of partners among those who had sex by end line – Total = 754; KwaZulu-Natal = 285; Mpumalanga = 469

^a^ Longitudinal analysis. Examined in generalized estimation equation (GEE) model, mixed-effect model and generalized linear latent and mixed model (GLLAMM). Focused on interaction variable between the intervention group and end line. Reported coefficients are from GLLAMM

^b^ Incidence outcomes (ever-sex, ever-pregnant, HSV-2, HSV-2/ever-pregnant) were examined in both survival analysis and logistic regression among those who did not have the outcome at baseline. Focused on the intervention group variable. Reported coefficients are hazard ratios from survival analysis

^c^ Logistic regression. Focused on the intervention group variable. Reported coefficients are odds ratios

^d^ Replaced with missing when the number reported was greater than or equal to 50

The ever-pregnant variable was positive and significant in the full sample and in KwaZulu-Natal; this effect was also positive in Mpumalanga but did not attain significance. This suggests that girls exposed in the intervention schools with the SLP were significantly more likely to be pregnant at end line than girls in the control schools. This was an unexpected result. Furthermore, while the estimated coefficient (i.e., hazard ratio) for HSV-2 in the full sample and in KwaZulu-Natal is less than one, it did not attain significance; this may reflect the size of the sample or a true nonsignificant result.

In the longitudinal sample, the girls from KwaZulu-Natal were significantly less likely to have initiated sex in the follow-up period than were the girls from Mpumalanga (Table 6). No other provincial difference was found to be significant in this sample; however, the estimated coefficients (i.e., odds ratio and hazard ratio) for the prevalence of HIV and the incidence of HSV-2, respectively, were both less than one, suggesting that in KwaZulu-Natal, the risk may be lower.

**Table 6:** Examination of differences between provinces (KwaZulu-Natal vs. Mpumalanga as reference group) among longitudinal girls between baseline and end line

Outcome	Full Sample
Ever sex^a^	0.52 (0.062(*p* < 0.001)
Ever pregnant^a^	1.05 (0.233) (*p* = 0.827)
End line HIV ^b^	0.77 (0.144) (*p* = 0.162)
HSV-2^a^	0.85 (0.176) (*p* = 0.424)
HSV-2 or ever-pregnant^a^	0.82 (0.138) (*p* = 0.232)

Reported figures are estimated coefficients (SE) and significance level

All models control for age group, orphanhood, if there is an HIV positive person in the household, food insecurity, religion, and districts

^a^ Incidence outcomes (ever-sex, ever-pregnant, HSV-2, HSV-2/ever-pregnant) were examined in both survival analysis and logistic regression among those who did not have the outcome at baseline. Focused on the dummy variable of KwaZulu-Natal. Reported coefficients are hazard ratios from survival analysis

^b^ Logistic regression. Focused on the dummy variable of KwaZulu-Natal. Reported coefficients are odds ratios

## Discussion

This evaluation used a rigorous study design with longitudinal data collection, random assignment of schools, and health status outcomes to determine whether the revised LO curriculum that used SLP and other supporting activities led to improved primary health status and behavioral outcomes as well as secondary outcomes such as knowledge, attitudes, self-efficacy, and HIV testing behaviors. The longitudinal analyses demonstrated that the only positive and significant effects of the intervention were on recent HIV testing behaviors in the full sample and in both provinces, and on recent SRH clinic visits among girls in Mpumalanga. We also found that girls in the intervention schools were significantly more likely to have reported experiencing an incident pregnancy compared to their counterparts in the control schools; this was an unexpected result. One possible explanation for this unexpected result may be that we had better follow-up in Mpumalanga and among intervention participants. If girls who became pregnant dropped out of school and were more likely to be lost to follow-up in control schools and in KwaZulu-Natal, this could lead to an underestimation of pregnancy events in those schools.

### Young people’s need for HIV prevention

The data from this study demonstrate continued need for targeting young people in South Africa with HIV prevention activities given the health status outcomes found here. We found that among the cohort of girls, who at baseline were on average 13.5 years old, about 3% had HSV-2 (2.2–2.6% in Mpumalanga and 3.6% in KwaZulu-Natal). Two years later, about 10% of the longitudinal cohort had HSV-2 (9.0–11.7% in Mpumalanga and 9.3–11.1% in KwaZulu-Natal)—with 7% of the sample having incident cases in the follow-up period. Of note, many girls in the cohort had never had sex and, therefore, this incidence (and prevalence) was particularly high among the subgroup of girls who reported being sexually experienced by end line (about 23% of the sample). The prevalence of HIV in this cohort (about 5%) reflects gaps in preventive services and care needs in KwaZulu-Natal and Mpumalanga. Our incidence and prevalence results from the biomarkers are comparable to results from high-school students in rural KwaZulu-Natal undertaken by Abdool Karim and colleagues [27]. In their sample of female students with a mean age of 16 years, HIV prevalence was 6.4% and HSV-2 prevalence was 10.7%.

### Continued gaps in understanding of what works

The lack of effect of the school-based life orientation curriculum on the incidence of HSV-2 or pregnancy, or prevalence of HIV suggests that factors beyond what young people learn in school influence sexual and reproductive health behaviors and outcomes. Future programs for young people in schools should consider strategies that engage community members, parents, and peer groups to help address issues that young people experience daily, including food insecurity, orphanhood, and living with HIV positive household members [15, 28, 29]. These may be distal but substantial factors that influence young people’s school attendance and sexual and reproductive health [29–31]. Pettifor and colleagues [32, 33], who evaluated a conditional cash transfer program in Mpumalanga, found that in both intervention and control schools, students who attained less than 80% school attendance were at greater risk of incident HIV or HSV-2; missing school may be linked to other risk taking behaviors outside the school environment. Thus, keeping girls in school is an important intervention for improved health and well-being and the life orientation curriculum that they are exposed to may be less important.

### Challenges with program implementation

The fact that there was no observed impact of the scripted lesson plans for the LO curriculum on the primary outcomes of the study raises the question of whether this was due to the program being ineffective as designed, as has been found in other school-based evidence-based programs or challenges with program implementation and lack of program fidelity [34]. In an assessment of national-level rollout of an evidence-based program in the Bahamas, Wang and colleagues [34] demonstrated that teachers taught a little more than half of the core activities and that the strongest predictor of fidelity of program implementation was the teachers’ comfort level with the program. Moreover, teachers who had more experience in the schools were less likely to implement the program with fidelity, while those who perceived the program to be important were more likely to implement it with fidelity. The authors also demonstrated that youth did not benefit from the program (e.g., self-reported knowledge and skills) if they received two or fewer sessions (out of eight). Further, in a qualitative assessment of the South Africa Life Orientation curriculum, Gavin and colleagues (2018) suggest that challenges at the individual, interpersonal, school, district, and community levels led to varying quality of program implementation by higher and lower resourced schools [35]. These types of challenges may have affected the roll-out of the program being evaluated here.

An alternative explanation for the lack of effect is that there was little difference in program exposure between the intervention and control participants which attenuated the results. In particular, since the scripted lesson plans were covering the same topics included in the standard (control) curriculum, the main difference between the arms was training of teachers on the new approach to delivery of the material and provision of SLP-related workbooks. Unfortunately, we were not able to obtain detailed records of program implementation at the classroom level to know whether the teachers in the intervention arm operationalized in the classroom the improved pedagogy skills they received training on or whether they consistently used the SLP-related workbooks. This would help to inform whether the teacher training had an effect on classroom implementation, or if teachers in both arms implemented the sexual and reproductive health content in a similar manner.

Another potential attenuating factor is that trained teachers from intervention schools may have been transferred to control schools. With the data available, this could not be monitored; however, any transferred teachers would not have had the relevant workbooks and materials to implement the program fully should this have happened. In follow-up discussions with Education Development Center, the implementing partner of the DBE program, we learned that there were numerous challenges with program implementation throughout the study period. First, because baseline data collection could not be collected until the third quarter of the school year, the program had already been implemented in intervention schools (prior to baseline data collection). This may have reduced the amount of change observed. Second, in the second year of the evaluation, when most of the cohort was in grade 9, we learned in retrospect that there was wide variability in implementation of the SLPs. About 41% of intervention schools in KwaZulu-Natal and 17% in Mpumalanga did not offer students any SLP lessons. In 6 and 17% of intervention schools in KwaZulu-Natal and Mpumalanga, respectively, all SLP lessons were implemented. Finally, the grade-10 curriculum (i.e., year three of exposure for those who were still in school) was not fully approved until late in the school year, which led to delayed implementation of the lessons in some schools (notably, some may have implemented the lessons prior to final approval). The evaluation team was asked to delay end line data collection by a couple of weeks to permit implementing some of the grade-10 lessons. Unfortunately, it was not clear if any or all lessons were implemented in the intervention schools in the grade-10 school year. Each of these factors contributed to the program not being implemented as intended at the time of study design and may have contributed to the null results.

### Limitations with evaluation

This evaluation study had several additional limitations that may have affected the results. We randomized schools to minimize the selection bias of schools and participants between the intervention and control arms; however, selection bias may still have been a concern if unobserved characteristics of students differed systematically between the participants and nonparticipants, and if these differences were related to the study outcome(s). For example, approximately 45% of eligible female participants for the longitudinal observation did not participate in some or all surveys throughout the study. First, girls who did not have written parental/guardian consent were not interviewed for the baseline study; these girls may be different from those who were able to get such consent. Second, there was 5% attrition of participants when they transferred or dropped out of schools. We attempted to minimize attrition by retrieving contact information from the baseline contact sheet and the baseline schools and following up with the girls at their home at midline and end line. We also applied an intention-to-treat analysis and analyzed data from girls based on their initial assignment to the LO program.

Social desirability bias may have led some participants to refuse to answer sensitive questions accurately. Gender norms may have also affected social desirability, including that the girls may have underreported their number of sex partners. Relatedly, the question on ever having been pregnant relied on self-reports, so the responses may not have been accurate. If the respondents’ reports were systematically different between the intervention and control arms, this would bias the results.

Finally, respondents may not have recalled their behaviors accurately. If the recall was not systematically different between the intervention and control arms, a measurement error would result, leading to a lower statistical power. If the recall was systematically different between the intervention and control arms, this also would bias the results.

## Conclusions

Based on the implementation data, the study team cannot conclude whether the DBE’s revision of the LO curriculum with SLPs and supportive activities is effective or not. That said, there are important lessons from this evaluation for future school based LO programs of this type. First, given that the DBE intends to scale-up the SLP, the program is not worth scaling up if implementation continues to be weak, as observed in the study period. Second, it is clear that girls in schools in grade 8 are at risk of HIV and STI, given the health status results presented here. These young people need skills-based programs offered through in- or out-of-school settings to help them avoid the risks of HIV, STI, and unintended pregnancies. The literature suggests that implementation of school-based lessons may not be enough; programs may need to also include access to HIV counseling and testing services in a youth-friendly manner, address gender norms and intimate partner violence, and address structural drivers that affect sexual and reproductive health behaviors and outcomes [16, 36, 37]. As part of this study, we attempted to partner with local-level HIV implementation partners to incorporate HIV counseling and testing services in study schools as a follow-up activity to the dried blood spot data collection. We faced policy and program barriers that will need to be addressed for programs seeking to provide in-school youth with the full range of needed HIV (and STI and unintended pregnancy) prevention services. Comprehensive and integrated programs for young people that include access to information and services in a youth-friendly setting have the strongest evidence of effect [36, 38], and the DBE should make these a priority to improve young people’s long-term health and well-being.

## Data Availability

Data used in this study are publicly available from the UNC Dataverse system. MEASURE Evaluation, 2019, “Impact Evaluation of a School-Based Sexuality and HIV-Prevention Education Activity in South Africa”, 10.15139/S3/SAQ9CQ, UNC Dataverse, V1.

## Abbreviations

CI: Confidence interval
DBE: Department of Basic Education
GLLAMM: Generalized linear latent and mixed models
HIV: Human immunodeficiency virus
HSV-2: Herpes simplex virus type 2
ICC: Intra-cluster correlation
LMIC: Low- and middle-income countries
LO: Life Orientation
ODK: Open Data Kit
PEPFAR: United States President’s Emergency Plan for AIDS Relief
RCT: Randomized controlled trial
SES: socioeconomic status
SLP: Scripted lesson plans
SRH: Sexual and reproductive health
STI: Sexually transmitted infections
USAID: United States Agency for International Development
USAID/SA: USAID Mission in South Africa

## Footnotes

1 At the time of baseline data collection, only 106 schools were included because of insecurity in some regions (*n* = 3), school-level refusal (*n* = 4), and two schools no longer including the relevant grades. Between baseline and midline, one school closed permanently so the end line number of schools was 105.

2 For the estimation of the incidence rate of HSV-2, we excluded from data analysis those who were HSV-2 positive at baseline. We assessed the HSV-2 status at baseline for those whose biomarker testing results were positive at end line. Those who acquired HSV-2 before study enrollment were excluded from data analysis at end line.

3 At the time of baseline data collection, we obtained written consent to store the samples for later HSV-2 testing. Baseline consent did not include permission for later HIV testing, which is why we only have prevalence of HIV rather than incidence.
